# Differences in foot self-care and lifestyle between men and women with diabetes mellitus**^1^**

**DOI:** 10.1590/1518-8345.1203.2761

**Published:** 2016-08-15

**Authors:** Mariana Angela Rossaneis, Maria do Carmo Fernandez Lourenço Haddad, Thaís Aidar de Freitas Mathias, Sonia Silva Marcon

**Affiliations:** 2 PhD, Assistant Professor, Departamento de Enfermagem, Universidade Estadual de Londrina, Londrina, PR, Brazil.; 3 PhD, Associate Professor, Departamento de Enfermagem, Universidade Estadual de Londrina, Londrina, PR, Brazil.; 4 PhD, Full Professor, Departamento de Enfermagem, Universidade Estadual de Maringá, Maringá, PR, Brazil.

**Keywords:** Diabetes Mellitus, Diabetes Complications, Diabetic Foot, Self Care, Nursing

## Abstract

**Objective:**

to investigate differences with regard to foot self-care and lifestyle between men and women with diabetes mellitus.

**Method:**

cross-sectional study conducted in a sample of 1,515 individuals with diabetes mellitus aged 40 years old or older. Poisson regression models were used to identity differences in foot self-care deficit and lifestyle between sexes, adjusting for socioeconomic and clinical characteristics, smoking and alcohol consumption.

**Results:**

foot self-care deficit, characterized by not regularly drying between toes; not regularly checking feet; walking barefoot; poor hygiene and inappropriately trimmed nails, was significantly higher among men, though men presented a lower prevalence of feet scaling and use of inappropriate shoes when compared to women. With regard to lifestyle, men presented less healthy habits, such as not adhering to a proper diet and taking laboratory exams to check for lipid profile at the frequency recommended.

**Conclusion:**

the nursing team should take into account gender differences concerning foot self-care and lifestyle when implementing educational activities and interventions intended to decrease risk factors for foot ulceration.

## Introduction

In Brazil, a total of 11.6 million people with diabetes mellitus (DM), aged between 20 and 79 years old, which corresponds to 8.7% of the 133.8 million individuals in this age range, was identified in 2014. DM is estimated to account for the death of 116,383 people in the same year, while 41.7% of these deaths occurred among individuals younger than 60 years old^1^.

Chronic complications accruing from DM include diabetic foot, the most common complication of type 2 DM (DM2), characterized by neurological, orthopedic, vascular and/or infectious alterations that precede the onset of non-healing wounds^2^. This complication is one of the main factors leading to the non-traumatic amputation of lower limbs. The global average rate of this type of mutilation among individuals with DM is estimated at 19.03%^3^.

Appropriate glycemic control, which demands changes in lifestyle and the adoption of self-care actions, is key to prevent diabetic foot and other complications^4^ ^-^ ^5^. These measures involve following a diet scheme, glucose and lipid profile monitoring, regular exercising, correct medication intake and foot care^2^. Dorothea Elizabeth Orem, a nurse, one of the first references for the practice of self-care, defines it as actions individuals initiate and perform by themselves to maintain life, health and wellbeing^6^. In her theory, Orem states that individuals unable to perform activities necessary to maintain their health present self-care deficit and require professional intervention^6^.

Limitations to implement changes in lifestyle and self-care actions that are required by the treatment are an issue widely known in the context of healthcare provided to diabetic individuals. Such limitations hinder the physiological response of individuals to the disease, the relationship between professionals and patients, also leading to increased direct and indirect costs^4^ ^,^ ^7^ ^-^ ^8^. The process of change is complex and the sex of patients has been identified as one of the factors interfering in the behavior and attitude of people who need to adopt new habits and implement self-care measures. Studies conducted among individuals with DM report that women present worse results concerning glycemic and lipid control^7^ ^,^ ^9^ while presenting worse behavior concerning foot care^10^ ^-^ ^12^. 

Even though the literature^7^ ^-^ ^12^ shows that differences between sexes are associated with the behavior of diabetic individuals towards the therapeutic plan, there are no Brazilian studies addressing differences related to lifestyle and self-care to prevent diabetic foot and other chronic complications. Therefore, this study's objective was to investigate differences in foot self-care and lifestyle between diabetic women and men.

## Method

 Cross-sectional study conducted with individuals with DM2, 40 years old or older, living in the urban area of a large city in the South of Brazil. To establish a representative and stratified sample per region, a prevalence of 11% of diabetic individuals in the population aged 40 years or older was considered^13^. First, we estimated the total population of diabetic individuals aged 40 years or older in the city's five regions (North, South, East, West and Center), and then we calculated the sample size for each region using Epi Info version 3.5.3, adopting a sampling error of 5% and 10% of losses in each of the city's regions. The sum of the samples calculated per region totaled 1,515 individuals. Then, we performed a new stratification of the sample per primary health care (PHC) service of each region. The study participants were drawn among those enrolled in the Hypertensive and Diabetics Individuals Registration System (HIPERDIA) of each service. Individuals with DM undergoing dialysis, who had active ulcers in the lower limbs, or those without cognitive capacity were excluded and replaced by the next in the draw. Such information was obtained from the medical files of the individuals selected in the draw and confirmed with the health teams of each PHC service. 

The individuals selected in the draw were invited to participate in the study through a telephone call or printed invitations received from the community health agents. Data were collected at PHC services. Those who failed to attend the date scheduled for data collection after three attempts, were not located at the address recorded in the medical file, or refused to participate in the study were replaced by another participant selected by a new draw until the sample was complete. 

An instrument developed by Bortoletto et al.^4^ ^)^ was adapted and used in this study. This tool addresses socioeconomic variables, lifestyle, clinical conditions, foot self-care and includes clinical examination of lower limbs. An interview was conducted with the participants to identify socioeconomic characteristics and variables related to lifestyle and foot self-care. The medical files were also consulted to verify variables that showed the patient's clinical conditions. After the interview, body weight and height were measured and the participants' lower limbs were clinically assessed. 

The variables of interest were those related to foot self-care and lifestyle of the individuals with diabetes. Foot self-care was assessed according to the interviewees' reports: dry between toes after shower (yes/no); regularly assess lower limbs (yes, no); regularly walk barefoot (yes, no); and perform foot scalding (yes, no). At the time of the interview, we also verified whether shoes were appropriate (yes, no), and whether hygiene was properly performed and toenails were properly trimmed (yes, no)^2^ ^,^ ^5^ ^,^ ^14^. Foot scalding consists in immersing the feet in a container with warm water. Hygiene was considered appropriate when the participant's feet were clean, dry, and with normal odor. Closed toe shoes with one extra centimeter in the internal part, made of soft leather or canvas/cotton, were considered appropriate^2^ ^,^ ^4^ ^-^ ^5^.

With regard to the variables concerning lifestyle, the participants were asked whether they followed an appropriate diet, exercised regularly (physical activity for at least 30 minutes more than three times a week), whether they smoked or consumed alcohol in excess (more than one dose per day for women and two daily doses for men). They were also asked whether they had regular laboratory exams to control DM. People diagnosed with DM are recommended to have their glycated hemoglobin (HbAc1) assessed at least once every six months and the lipid profile once a year^2^. The lipid profile consists of assessing triglycerides, total cholesterol, low-density lipoproteins (LDL) and high-density lipoproteins (HDL)^2^. Body weight and height were also verified and the body mass index (BMI) was determined: BMI ≥ 25 kg/m^2 (^ ^2^ was considered overweight.

Socioeconomic variables included sex, age, marital status, self-reported race, education, and socioeconomic status according to ABEP (Brazilian Association of Survey Companies)^15^. Socioeconomic status was classified into: classes A and B, class C, and classes D and E. 

The participants' clinical condition was assessed according to the variables: time since diagnosis, type of treatment, and presence of hypertension or chronic complications accruing from DM. The presence of chronic complications was established when the individual presented: diabetic retinopathy, diabetic nephropathy, stroke, acute myocardial infarction (AMI), peripheral diabetic neuropathy, and peripheral vascular disease and/or foot deformities. Changes in the lower limbs were verified through clinical examination of the feet in accordance to the American Diabetes Association's guidelines^2^.

Prevalence ratio (PR) was used for the analysis of association. The Wald Chi-square test was performed with a level of significance of 5% to determine whether there were differences between men and women with regard to variables related to lifestyle, laboratory exams according to the recommended schedule and foot self-care. Afterwards, these analyses were adjusted according to socioeconomic and clinical variables, using Poisson Regression models. Thus, variables that presented p<0.20 in the bivariate analysis with lifestyle and foot self-care variables were selected. These variables were selected to adjust the model, considering that diabetes mellitus is associated with people's living conditions and aggravates over time according to the patients' clinical aspects. 

The female sex was established as reference because recent studies have identified that men more frequently present DM-related self-care deficit when compared to women^7^ ^,^ ^10^ ^,^ ^12^. Statistical analysis was conducted using Statistical Package for the Social Sciences 21.0. This study received approval from the Institutional Review Board at the State University of Londrina, in accordance to CAAE (Certificate for Ethical Assessment) 0123.0.268.268-11.

## Results

A total of 954 (63.0%) out of 1,515 interviewees were women. Age ranged between 40 and 84 years old, average of 65 years, median of 66 years. Men's median was 66 years and women's median was 65 years old. Most individuals, regardless of sex, reported being Caucasian: 50.6% were women and 58.6% were men. Most had less than eight years of education: 78.4% of women and 66.7% of men. Regarding socioeconomic status, 62.8% of women were in class C, followed by 23.0% in Classes A/B and 14.2% in Classes D/E, while 32.3% of men were in classes A/B, 59.0% in class C, and only 8.7% were in classes D/E. 

Regarding clinical conditions, more than half of the participants received the diagnosis less than 10 years earlier. Of these, 54.1% were women and 52.6% were men. The prevalence of insulin was similar among men and women (23.8% in women and 22.8% in men), as was the prevalence of hypertension; both sexes presented high rates of hypertension (81.6% in women and 76% in men). 

In terms of lifestyle, regular exercise was more frequent among men (25.0%) than among women (19.9%), while following an appropriate diet was more frequent among women (77.3%). Both men (83.6%) and women (87.8%) were overweight or obese, while smoking or drinking alcohol in excess was more prevalent among men, 11.8% and 37.4% respectively; only 6.5% of the women reported smoking and 11.4% reported the consumption of alcohol beyond the recommended. Undergoing laboratory exams as regularly as recommended to control DM was more frequent among women: 40.8% checked HbAc1 in the last six months and 51.8% collected exams to check the lipid profile in the last year, compared to 34.0% and 41.5% among men, respectively (Table 1). 

**Table 1:** Distribution of lifestyle-related variables of people with diabetes mellitus according to sex. PR, Brazil, 2012.

Variables		Female (954)	Male (561)
		n (%)	n (%)
Controlled diet			
	Yes	737 (77.3)	370 (66.0)
	No	217 (22.7)	191 (34.0)
Regular exercise regular			
	Yes	190 (19.9)	140 (25.0)
	No	764 (80.1)	421 (75.0)
Smoking			
	No	892 (93.5)	495 (88.2)
	Yes	62 (6.5)	66 (11.8)
Excessive consumption of alcohol			
	No	845 (88.6)	351 (62.6)
	Yes	109 (11.4)	210 (37.4)
BMI within normal parameters			
	Yes	116 (12.2)	92 (16.4)
	No	838 (87.8)	469 (83.6)
Glycated hemoglobin in the last 6 months			
	Yes	389 (40.8)	191 (34.0)
	No	565 (59.2)	370 (66.0)
Lipid profile in the last year			
	Yes	494 (51.8)	233 (41.5)
	No	460 (48.2)	328 (58.5)

Regarding foot self-care, women more frequently performed the care necessary to prevent ulcerations, though men presented better habits concerning appropriate shoes (62.0%). Only 10.7% of men reported feet scalding, while 40.4% of the women admitted the practice (Table 2).

**Table 2:** Distribution of foot self-care related variables of people with diabetes mellitus according to sex. PR, Brazil, 2012.

Variables		Female (954)	Male (561)
		n (%)	n (%)
Dried between toes after bath			
	Yes	799 (83.8)	403 (71.8)
	No	155 (16.2)	158 (28.2)
Checked feet regularly			
	Yes	636 (66.7)	320 (57.0)
	No	318 (33.3)	241 (43.0)
Feet scalding			
	No	664 (69.6)	501 (89.3)
	Yes	290 (40.4)	60 (10.7)
Walked barefoot			
	No	722 (75.7)	371 (66.1)
	Yes	232 (24.3)	190 (33.9)
Used proper shoes			
	Yes	199 (20.9)	348 (62.0)
	No	755 (79.1)	213 (38.0)
Properly trimmed nails			
	Yes	469 (49.2)	139 (24.8)
	No	485 (50.8)	422 (75.2)
Maintained proper hygiene of feet			
	Yes	877 (91.9)	478 (85.2)
	No	77 (8.1)	83 (14.8)

The Poisson regression analysis, used to identify potential differences between sexes regarding lifestyle and foot self-care, showed statistically significant differences with regard to self-reported diet control; men presented deficit in this aspect (PR=1.47/ CI95%=1.22-1.77). Men also failed to regularly take laboratory exams to check the lipid profile in the last year (PR=1.15/ CI95%=1.06-1.27). These results were found after adjusting for socioeconomic and clinical variables of DM, smoking and excessive alcohol consumption (Table 3). 

Additionally, all foot self-care related variables were statistically associated with the patient's sex, even after adjusting for socioeconomic and clinical variables, smoking and alcohol consumption. Men presented greater deficits in the following: did not dry between toes after shower (PR=1.75/CI95%=1.43-2.15), did not regularly check feet (PR=1.32/CI95%=1.15-1.51), frequently walked barefoot (PR=1.31/CI95%=1.10-1.55), inappropriately trimmed nails (PR=1.49/CI95%=1.37-1.62) and presented inappropriate hygiene (PR=1.49/CI95%=1.37-1.62). Nonetheless, being a male appeared statistically associated with lower prevalence of self-care deficit regarding wearing appropriate shoes (PR=0.49/CI95%=0.44-0.55) and not performing feet scalding (PP=0.33/CI95%=0.25-0.44) when compared to women (Table 3).

**Table 3:** Poisson Regression models for self-care deficit concerning lifestyle that does not favor the control of diabetes mellitus and foot self-care among men compared to women. PR, Brazil, 2012.

Variables		Raw PR (CI 95%)	p-value	Adjusted PR (IC 95%)*	p-value
Lifestyle					
	Did not control diet	1.49 (1.27-1.76)	<0.001	1.47 (1.22-1.77)	<0.001
	Did not exercise regularly	0.93 (0.88-0.99)	0.026	0.96 (0.90-1.02)^†^	0.193
	BMI above normal parameters	0.95 (0.91-0.99)	0.022	0.96 (0.91-1.00)^‡^	0.800
	Checked glycated hemoglobin more than 6 months ago	1.11 (1.02-1.20)	0.008	1.06 (0.97-1.16)^§^	1.470
	Checked lipid profile more than one year ago	1.21 (1.10-1.33)	<0.001	1.15 (1.06-1.27)^||^	0.007
Foot self-care					
	Did not dry between toes after shower	1.73 (1.42-2.10)	<0.001	1.75 (1.43-2.15)^¶^	<0.001
	Did not check feet	1.28 (1.13-1.46)	<0.001	1.32 (1.15-1.51)**	<0.001
	Performed feet scalding	0.35 (0.27-0.45)	<0.001	0.33 (0.25-0.44)^††^	<0.001
	Walked barefoot	1.39 (1.18-1.63)	<0.001	1.31 (1.10-1.55)^‡‡^	0.002
	Used inappropriate shoes	0.48 (0.42-0.53)	<0.001	0.49 (0.44-0.55)^§§^	<0.001
	Inappropriately trimmed nails	1.48 (1.36-1.60)	<0.001	1.49 (1.37-1.62)^||||^	<0.001
	Presented poor feet hygiene	1.83 (1.36-2.45)	<0.001	1.98 (1.46-2.67)^¶¶^	<0.001

*Adjusted according to: socioeconomic and clinical variables, smoking and excessive alcohol consumption.

†,‡,||,§ Not adjusted for marital status.

¶,**,††,|||| Not adjusted for race.

§§ Not adjusted for education.

†,¶,††,‡‡ Not adjusted for type of treatment.

†† Not adjusted for overweight/obesity.

¶,**,§§ Not adjusted for smoking.

¶,**,¶¶ Not adjusted for excessive alcohol consumption.

## Discussion

This study revealed that most practices related to changes in lifestyle that are required to properly control DM and foot self-care, to prevent ulceration, are associated with gender. Proper diet was more prevalent among women, as reported in another study conducted with 4,839 people with DM, in which women reported a more frequent daily consumption of fruits and vegetables and low intake of fatty foods, when compared to men^10^. 

In contrast, being a man was associated with a higher prevalence of regular exercise. This result was also verified in other studies in which women with DM tended to be less physically active than men(7-8), while men were less adherent to recommended diet(7,16) and glycemic monitoring^7^. 

Regarding the control of DM by taking laboratory exams to check for glycated hemoglobin and lipid profile, men presented a higher prevalence of deficit with regard to this aspect. This result may be associated with the fact that women are more attentive to symptoms and physical signs of diseases and more frequently seek health services than men^8^ ^,^ ^10^. Additionally, other studies identified that women present a higher prevalence of changes in the lipid profile and therefore require medication to treat dyslipidemia early^2^ ^,^ ^7^ ^,^ ^9^. 

With regard to diabetic foot self-care, men presented greater deficit comparing to women. A prospective study with seven years of follow-up identified that being a male was a risk factor for amputation among patients with diabetic feet, together with other factors such as long time since diagnosis, high glycated hemoglobin, retinopathy and the use of insulin^17^. 

Another important aspect verified in this study refers to the fact that women present greater prevalence of foot self-care, such as drying between toes after shower, checking feet regularly, properly trimming nails to avoid lesions and ingrown toenails, not going barefoot, and performing proper hygiene. These actions suggest women acquired knowledge about self-care necessary to prevent ulceration in the lower limbs. Drying between toes after shower and performing proper hygiene of feet decrease the risk of bacterial and fungal infections, some of the main conditions that precede the amputation of lower limbs^4^ ^,^ ^12^ ^,^ ^14^.

Men less frequently presented deficits related to feet scalding and inappropriate use of shoes. Feet scalding is more frequent among women because it is related to the aesthetic habit of skin exfoliation and cuticles removal. Additionally, culturally, women wear shoes of different models that include high heels, side and heel vents, which expose toes. Another hypothesis is related to the fact that individuals may not have access to more appropriate shoes, given a lack of financial resources. A case control study analyzing factors associated with amputations among individuals with DM verified that people who did not wear appropriate shoes were 4.75 times more likely to suffer an amputation that those who did^18^. 

Note that most of the interviewees were elderly individuals with a low educational level and predominantly belonged to economic Class C. It is a fact that socioeconomic conditions are directly related to risk factors leading to complications of Non-Transmissible Chronic Diseases (NTCD), as the lack of financial resources interferes in the access to healthcare services, options of treatments, and hinders the adoption of preventive measures, necessary to avoid amputations^2^ ^,^ ^19^. In her theory, Orem states that the commitment of people to perform self-care practices depends on cultural and educational aspects, individual skills and limitations, together with experience of life, health condition and availability of resources^6^. 

In this sense, socioeconomic factors interfere in the lifestyle and self-care practices of individuals with DM, especially with regard to understanding orientations on how to manage the disease and resources necessary to lead a healthy life. Low education and limitations concerning social and financial conditions pose a challenge to healthcare workers because different strategies to provide self-care education are required^2^ ^,^ ^4^ ^,^ ^12^.

Nursing consultations and home visits implemented during the follow-up of individuals with DM within the PHC service favor the identification of self-care deficits, the ability of individuals to perform care, and the family network support available to patients. These meetings enable workers to monitor self-care actions or actions performed by a caregiver, and manage factors interfering in the development of skills^20^. 

Still with regard to the nursing consultation, examining lower limbs and stratifying the individual's risk for developing ulcers is key to prevent and treat diabetic feet^2^. A study assessing the effectiveness of a nursing care program delivered to individuals with DM was conducted during two years and verified that this strategy decreased the risk of ulceration in the participants' lower limbs and prevented the emergency of new ulcers among those with a history of previous lesion. The care measures that were implemented during the program included regular assessment of feet, self-care education, treatment of ulcers and mycoses, skin hydration, and referral to medical specialties for the more severe cases, among others^21^. 

Healthcare workers have to pay attention to these aspects in an attempt to identify clinical changes and the care needs of patients to control DM, prevent chronic complications, and treat diabetic feet early on^2^ ^,^ ^14^. Note that orientation regarding the therapeutic plan and self-care education needs to take into account that men and women hold different beliefs regarding the benefits of DM-related self-care. Other studies^16^ ^-^ ^17^ verified that women present better education in DM and higher expectations regarding the benefits accruing from self-care, while men are usually reluctant to acknowledge their health problems and seek professional care. 

Another study addressing male self-perceived health verified that most men, even after receiving the diagnosis of a chronic disease, did not seek medical care, mainly alleging lack of time due to working days, incompatibility between their schedules and the health services' functioning hours, lack of severe symptoms, or because they faced more difficulty to access healthcare services than women^16^. With regard to women with DM, the study identified that they needed greater professional and familial support to adhere to the treatment than men. Lack of support also influenced the adoption of a proper diet because women were not willing to cook foods, necessary for their diet, but which their families did not appreciate, such as restricted salt and sugar. Additionally, those women facing financial hardship consider it selfish to buy foods that exclusively meet their needs^8^.

Therefore, some cultural, social and behavioral aspects permeate the lifestyle and self-care of men and women with DM and PHC workers cannot ignore these results when devising a therapeutic plan and care support. Healthcare workers often judge patients and their families, labeling them as careless or irresponsible for not following recommendations without knowing the context of these individuals. Establishing bonds and properly welcoming the needs of people diagnosed with NTCD should be the first step towards intervening in risk factors and managing these diseases. Monitoring individuals with DM, aiming not only to provide treatment but also to improve the quality of life of these individuals, requires professionals to have a holistic approach towards intervenient issues in the adherence to treatment and self-care education. 

It is worth noting that the health of men and the aspects involved in the management of their health is a challenge for healthcare workers who need strategies that enable these patients to more frequently seek care and adhere to NTCD prevention and control measures. 

## Conclusion

This study's limitations are related to self-reported information concerning self-care practices and lifestyle and, for this reason the diagnosis of chronic complications may be underestimated while eating habits, exercise, and foot self-care may be overestimated. Additionally, it is not possible to establish a relationship of cause and effect between dependent and independent variables due to this study's cross-sectional design. There are also differences related to sociocultural characteristics of different populations, which play an important role in influencing the behaviors of individuals toward a disease and could be better addressed using qualitative methods. 

The results show statistically significant differences regarding lifestyle and self-care practices of men and women with DM. The population under study presented deficit in self-care necessary to prevent DM chronic complications. From this perspective, we believe that the analysis performed in this study concerning the behavior of men and women with DM with regard to self-care and lifestyle contributes to the planning and implementation of care provided to individuals with NTCD, in order to decrease mortality indexes and impairment that results from DM complications. 
